# A Preliminary Study on Discriminant Analysis of Syndrome Types in the Recovery Period of Stroke in Traditional Chinese Medicine

**DOI:** 10.1155/2018/6079595

**Published:** 2018-12-04

**Authors:** Zhichao Gan, Dongxue Zhang, Zhihui Huang, Lanrong Chen

**Affiliations:** ^1^College of Humanities and Management, Fujian University of Traditional Chinese Medicine, 350122, China; ^2^Third Department of Brain Disease, Affiliated Rehabilitation Hospital of Fujian University of Traditional Chinese Medicine, 350003, China

## Abstract

**Objective:**

The purpose of this study is to understand the distribution of syndrome types, the data of four diagnostic information variables, and the correlative degree and diagnostic value of four diagnosis indexes and syndromes in patients with the recovery period of ischemic stroke through clinical case data.

**Methods:**

This study developed a unified clinical case collection table, following the clinical research design, measurement, and evaluation methods, using Chi-Square test, logistic regression analysis, and diagnostic test evaluation methods for data screening, analysis, and testing.

**Results:**

According to the comprehensive comparison, analysis, and evaluation, the study concluded that the specificity, sensitivity, positive likelihood ratio, positive predictive value, negative predictive value, accuracy, and Youden index of “thick fur, slippery pulse” was the highest.

**Conclusion:**

“thick fur, slippery pulse” is the best combination to diagnose the phlegm-stasis in channels during the recovery period of ischemic stroke.

## 1. Introduction

Stroke [1, 2], is a Traditional Chinese Medicine (TCM) disease name [3, 4], the earliest from the “Huangdi Neijing”. Its clinical manifestations are suddenly faint, hemiplegia, sluggish speech, and tongue skew. It is characterized by acute illness and rapid change, just like the wind. The World Health Organization (WHO) defines strokes as caused by the interruption of the blood supply to the brain, usually because a blood vessel bursts or is blocked by a clot. This cuts off the supply of oxygen and nutrients, causing damage to the brain tissue [5]. There are also numerous reports and authoritative statistics confirming that China has become a high-level country for cerebrovascular disease [6–8]. As one of the most highly developed cerebrovascular diseases in China, the Chinese government has made various measures to prevent and control stroke, including policies [9, 10], guidelines, clinical pathways, and treatment programs [11]. Not only is stroke appreciated in China, but it is one of the diseases that cause great concern worldwide. The WHO has included prevention of stroke as one of the 9 voluntary global targets achieved 2025 years ago [12].

Therefore, this study chooses the stroke disease as the research object. We hope to make use of the advantages of the Traditional Chinese Medicine, according to the four diagnostic information variables of the Traditional Chinese Medicine to establish a model for the classification of stroke syndrome type. Moreover, the model is embedded in the Traditional Chinese Medicine clinical pathway system for verification. It will lay a theoretical and data foundation for the study of auxiliary diagnosis and early warning of stroke disease.

## 2. Materials and Methods

In order to explore the relationship between the four diagnostic information variables of TCM and the recovery period of ischemic apoplexy, this study collected 420 cases of ischemic stroke during the recovery period of clinical case data. The case data used by the institute were obtained with the knowledge and consent of the patients and their families and allowed to be used after approval by the hospital ethics committee.

### 2.1. Materials

#### 2.1.1. Inclusion Criteria

If the case data meets all of the following conditions, the case category of this study is included.

According to the “Guidelines for the Diagnosis and Treatment of Common Diseases in the Traditional Chinese Medicine” [13] and the “Guidelines for the Diagnosis and Treatment of Acute Ischemic Stroke in China (2014 Edition)” [14] (see Table 1), it is confirmed that the first diagnosis of the patient is the stroke disease (TCD Code: BNG080; ICD-10 Code: I63).

The case belongs to the recovery period. See Table 2.

#### 2.1.2. Exclusion Criteria

If the case data meets one of the following conditions, the case category of this study is not included. See Table 3.

### 2.2. Methods

**Figure 1:**
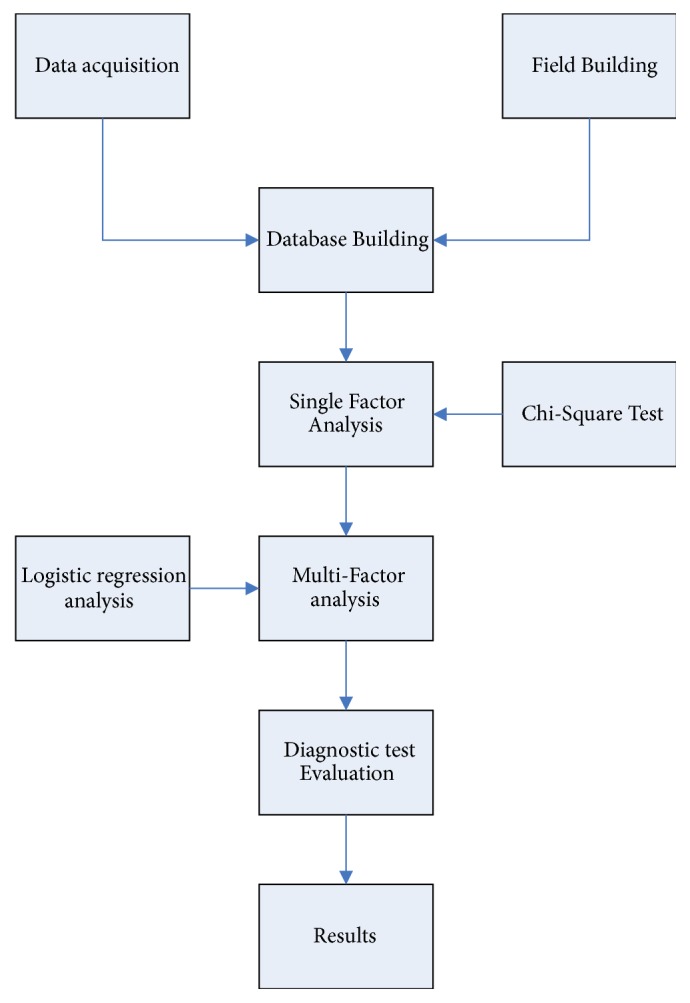
Research flowchart.

Data were analyzed using the SPSS 16.0 in this study. Before the data is analyzed, we check the accuracy of the data. The Chi-Square test is a non-parametric test method, which can be used to analyze the correlation of two categorical variables [15, 16]. So, first of all, we use this method to select the four diagnostic information variables of the TCM in the clinical case data of ischemic stroke recovery period. Logistic regression analysis model is often used in epidemiology to explore the risk factors of a disease and to predict the probability of disease occurrence [17]. Secondly, this study used the above methods to explore the high-risk variables of disease occurrence in the clinical case data of ischemic stroke during the recovery period. At last, we use the diagnostic test to evaluate the high-risk variables and determine their accuracy. The technical route is shown in Figure 1.

The calculation formula of Chi-Square test is as follows.

(1) X2=∑A−T2T

(2) V=R−1C−1

The logistic regression analysis formula is as follows.

(3) logitp=ln⁡p1−p=b0+b1X1+b2X2+⋯+bmXm

The evaluation index of diagnostic test is calculated as follows.

(4) Se=aa+c×100%

(5) Sp=db+d×100%

(6) Accuracy=a+dn×100%

(7) YI=Se+Sp−1

(8) LR+=a/a+cb/b+d=Se1−Sp

(9) LR−=c/a+cd/b+d=1−SeSp

(10) PV+=aa+b×100%

(11) PV−=dc+d×100%

## 3. Results and Discussion

### 3.1. Results

#### 3.1.1. Baselines

**Figure 2:**
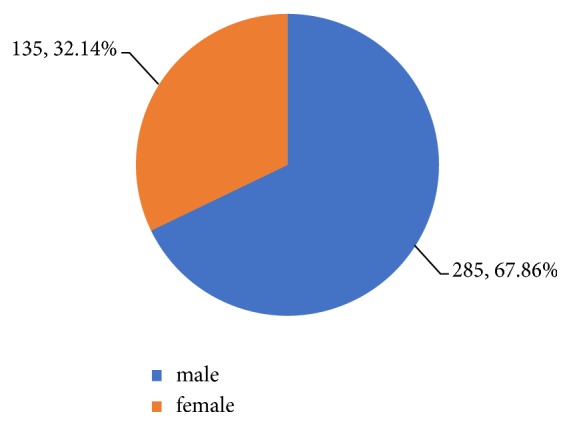
Percentage of gender distribution.

*Gender Grouping*. In 420 cases, there were 285 cases of male patients and 135 cases of female patients. The gender ratio is about 2.11:1. The gender distribution of the sample is shown in Figure 2.

**Figure 3:**
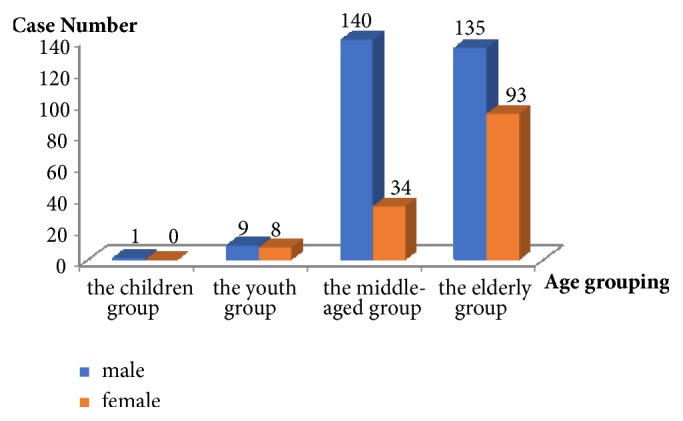
Age group distribution column chart.

*Age Grouping*. According to the general consensus of China on the classification of age groups, the study divides 420 case data into four groups, namely, the children group (younger than 18 years old), the youth group (between the age of 18 to 40 years old), the middle-aged group (between 41 years of age and 65 years old), and the elderly group (older than 65 years). The number of groups for each age is shown in Figure 3. What is more, the average age of data in 420 cases was 65.34 ± 14.16 years, the minimum age was 8 years, and the maximum age was 98 years.

*Syndrome Type Grouping*. According to March 2017, the Chinese Medicine Administration of China revised the TCM clinical pathway and the TCM treatment plan of 92 diseases (2017 Edition); the recovery period of ischemic stroke is divided into four kinds of syndromes, respectively: Qi deficiency and blood stasis syndrome, phlegm-stasis in channels, wind formation from yin deficiency syndrome, phlegm-heat fu-organ sthenia syndrome. In the data of 420 cases, there were 248 cases of the phlegm-stasis in channels, 120 cases of the Qi deficiency and blood stasis syndrome, and 52 cases of the wind formation from yin deficiency syndrome. The types of syndromes are distributed as shown in Table 4.

#### 3.1.2. Single Factor Analysis Results

Because of the 420 cases of clinical cases, the ratio of the phlegm-stasis in channels is 59.05%, so the syndromes of all samples are classified as the phlegm-stasis in channels and the non-phlegm-stasis in channels. In the sample database, the type of syndrome is reassigned, the case of the phlegm-stasis in channels is 1, and the case of the non-phlegm-stasis in channels is 0. According to the statistical calculation, the 420-sample data contains 86 signs variables. After the Chi-Square test method was calculated, at the *α* = 0.05 level, a total of statistically significant signs were selected for 28, the results are shown in Table 5.

#### 3.1.3. Multifactor Analysis Results

On the basis of the results of the single factor analysis, the high-risk variables of the phlegm-stasis in channels in the recovery period of ischemic stroke were obtained by using the logistic regression analysis method based on syndrome type as the dependent variable. Using the method of binary logistic regression, the selection of variable by forward LR stepwise method was adopted, and 7 statistically significant variables were obtained under the condition of *α*_Entry_ = 0.05, *α*_Removal_ = 0.10, that is, mental weakness, languid, distortion of commissure, body emaciation, limb without autonomous activity, thick fur, and greasy fur. The results are shown in Table 6.

As can be seen from Table 6, the variable “thick fur” of the “odds ratio” is 12.828 and the variable “greasy fur” of the “odds ratio” is 3.928. It means that the two variables of “thick fur” and “greasy fur” can effectively distinguish whether the case is a syndrome of the phlegm-stasis in channels. The “odds ratio” of variables, including “mental weakness”, ” languid”, ” distortion of commissure”, ” body emaciation”, and “limb without autonomous activity”, is less than 1, so it can be explained that these variables have little influence on whether or not they belong to the phlegm-stasis in channels.

#### 3.1.4. Diagnostic test Evaluation Analysis Results

Multivariate analysis was used to obtain the variable index of the risk of the phlegm-stasis in channels in patients with stroke, combined with clinical experience of Traditional Chinese Medicine. It is believed that the diagnosis of the phlegm-stasis in channels during the recovery stage of stroke has a large number of diagnostic indexes, which are considered to be 6 indexes of “thick fur”, “greasy fur”, “stringy pulse”, “slippery pulse”,” purplish tongue”, and “hesitant pulse”. By using the method of diagnostic experiment, the 6 indexes were combined statistically and analyzed, and the diagnostic value of the combined results of each index was screened by comparing the results of Traditional Chinese Medicine clinical diagnosis. The results are shown in Tables 7 and 8.

Table 7 lists the meaningful 24 combinations of 6 indicators. From the point of view of specificity, 16 different combinations of the specificity of the situation reached 99.42%. From the level of sensitivity, there are 7 combinations of the sensitivity of more than 60%, of which the highest sensitivity is the “slippery pulse” of a single indicator; its sensitivity is 91.53%, followed by the “stringy pulse” and the “greasy fur”, sensitivity of 83.47% and 83.06%, respectively. In the positive predictive value, except the positive predictive value of the “hesitant pulse” which is 4.55%, the other combinations are more than 70%, and the positive predictive value of the “thick fur” is the highest, 97.73%. In the negative predictive value, more than 60% of the combination has 7, with the “slippery pulse” having the highest negative predictive value, 82.93%. In terms of the authenticity of diagnostic tests, it is necessary to consider the two items of accuracy and Youden index. The accuracy and Youden index of the “greasy fur” are the highest, respectively, 78.81% and 0.5574. Overall, through comprehensive analysis and contrast sensitivity, 8 evaluation contents, such as specificity, accuracy, Youden index, positive likelihood ratio, negative likelihood ratio, positive predictive rate, and negative predictive rate, can be considered as the optimal combination of the phlegm-stasis in channels during the recovery period of stroke by the combination of the “thick fur and slippery pulse”.

### 3.2. Discussion

The phlegm-stasis in channels is one of the most common syndromes in the recovery period of ischemic stroke. In the theory of TCM [18], the phlegm and the blood stasis are the main pathogenesis of ischemic stroke. “Herbal New” [19] records that No stroke patient is not caused by the phlegm-stasis in channels. From the disease course development point of view, the main manifestation of stroke is the phlegm and the blood stasis in the meridians, leading to tendons lost in warm. The relevant terminology of TCM is explained in Table 9. From the research results of many scholars at home and abroad on the syndromes of the phlegm-stasis in channels in the recovery period of ischemic stroke, because of the characteristics of flexibility and objectivity in TCM syndrome differentiation, the dialectical result is different because of the difference in the focus of dialectical evidence. Xingping Zhang [20], through the collection of ancient and modern medical treatment of the phlegm-stasis in channels of the idea and method, reported that the phlegm-stasis in channels can be reflected from the tongue image, mainly manifested in the white tongue, tongue coating thick characteristics. Jingren Zhang [21], from his more than 70 years of clinical experience in TCM, summarized the diagnosis and treatment of the phlegm-stasis in channels. He thought the tongue color is the purple dark and the greasy moss is the distinguishing main characteristic of the phlegm-stasis in channels. Baohua Li [22] believes that the phlegm and the blood stasis are the main cause of stroke. It mainly shows the tongue color is purple dark and the greasy moss in clinical symptoms.

From the theory of the Western medicine, the appearance of the “thick fur” means that the digestive function of the stomach is problematic. Because the stomach digestion function is insufficient, food cannot completely decompose. These substances, which cannot be absorbed by the body, then form “phlegm” that hinders the normal functioning of other system functions of the human body. Therefore, the clinical manifestation of hemiplegia, tongue skew, speech unfavourable, dizzy, and cough phlegm includes many and sticky symptoms. In addition, “slippery pulse” is one of the common pulses of TCM. From the electrocardiogram, only the heart rate of the slippery pulse is generally more than 80 times per minute [23]. The most prominent feature of the slippery pulse is reflected in the TCM pulse diagnosis, showing a string of beads from the fingertips across the feeling.

However, there are some limitations in this study. From the point of view of syndrome pattern distribution, in the clinical case data of all stroke recovery periods, the distribution of syndromes showed partial distribution, no cases of the syndrome of excessive fu-viscera caused by phlegm-heat were found, and the cases with the Qi deficiency and blood stasis syndrome and the wind formation from yin deficiency syndrome were few, so the phlegm-stasis in channels with more cases was selected as the classification variable, and other syndromes were not statistically analyzed. From the collected case data, because the various variables are classified by two classification methods for qualitative labeling, we did not make further grades or quantitative provisions, so in the type of the phlegm-stasis in channels analysis we cannot carry out scientific quantitative analysis to determine the severity of the disease.

## 4. Conclusions

Through retrospective investigation and analysis of clinical case data, using the Chi-Square test, logistic regression analysis, and diagnostic test evaluation method, the identification basis of syndrome of the phlegm-stasis in channels during the recovery period of ischemic stroke was studied. The “thick fur, slippery pulse” is the best combination to diagnose whether the syndrome type of patients with ischemic stroke is the phlegm-stasis in channels. It can provide some reasonable thinking and reference method for early warning and identification of ischemic stroke. In the future research, we will gradually improve the problems in the collection of case data to provide a solid foundation for the early warning and identification of the syndrome of stroke.

## Figures and Tables

**Table 1:** Chinese and Western Medicine diagnostic standards table.

Diagnostic criteria for the Traditional Chinese Medicine	Diagnostic criteria for the Western Medicine
(1) The clinical manifestation of stroke is unconsciousness, hemiplegia, tongue skew, sluggish speech, or headache, dizziness, drinking water cough, unsteadiness of walking and so on.	(1) The onset nature of stroke is acute.
(2) It is often acute onset in a quiet state, and the disease gradually increases. A small number of patients have a sudden onset and the disease develops rapidly, often accompanied by unconsciousness.	(2) In most cases, a stroke causes a local neurological impairment, which in a few cases results in a complete neurological impairment.
(3) Most stroke patients have an inducement before the onset, often with signs of warning. For example, in one day or a few days of dizziness, headache, tinnitus, short-term speech vague, limb numbness.	(3) If the results of imaging examination can determine the presence of ischemic lesions, the patient's clinical symptoms or signs duration is not limited. If the results of imaging examination cannot determine the presence of ischemic lesions, the patient's clinical symptoms or signs must be longer than 24 hours.
(4) The majority of patients are older than 40 years of age.	
(5) With the above clinical manifestations, combined with the form of disease, inducement, symptoms, and age, stroke disease can be diagnosed. The diagnosis of ischemic stroke can be identified by combining imaging examinations (head CT or MRI).	

**Table 2:** Ischemic stroke staging table.

Stage	Description
Acute stage	Stroke onset within 2 weeks. If the patient appears unconscious, the time can be extended to 4 weeks of onset.
Recovery period	Stroke onset within 2 weeks to 6 months.
Sequela period	6 months after the onset of stroke.

**Table 3:** Exclusion standards table.

No	Description
1	The case belongs to the Transient Ischemic Attack (TIA).
2	The examination was made to diagnose the cerebral embolism caused by brain tumors, brain trauma, brain parasites, metabolic disorders, rheumatic heart disease, coronary heart disease and other heart diseases combined with atrial fibrillation.
3	Cases are caused by bleeding disorders or clotting dysfunction.
4	Cases with severe lung infections, liver, kidney, digestive system or endocrine systems diseases and other systemic diseases, and the condition is more serious and causes other diseases to replace the main disease of stroke.
5	Due to the wishes of patients and their families or other factors cannot be combined to complete the study of clinical data collected.
6	The case belongs to the acute period or sequela period.

**Table 4:** Distribution results of case data in the recovery period of stroke.

Syndrome Type	Frequency	Percentage
phlegm-stasis in channels	248	59.05%
phlegm-heat fu-organ sthenia syndrome	0	0%
wind formation from yin deficiency syndrome	52	12.38%
Qi deficiency and blood stasis syndrome	120	28.57%

**Table 5:** The Chi-square test results for 86 variables.

Variable	Value	P
Instability of gait	4.205	0.040
Mental weakness	30.034	<0.001
Languid	16.033	<0.001
Distortion of commissure	6.624	0.010
Flush face	19.343	<0.001
Dim complexion	20.806	<0.001
Pallid complexion	15.385	<0.001
Palpitation	5.034	0.025
Body emaciation	6.474	0.011
Inarticulateness	4.548	0.033
Soreness and weakness of waist and knees	39.822	<0.001
Faint low voice	12.403	<0.001
Limb weakness	6.456	0.011
Limb without autonomous activity	4.531	0.033
Deep red tongue	9.954	0.002
Purplish tongue	25.372	<0.001
Pale tongue	25.437	<0.001
Red tongue	12.375	<0.001
Thin fur	132.057	<0.001
Thick fur	30.409	<0.001
Greasy fur	131.738	<0.001
Few fur	36.702	<0.001
White fur	24.134	<0.001
Deep pulse	9.401	0.002
Thready pulse	161.898	<0.001
Slippery pulse	126.732	<0.001
Hesitant pulse	60.375	<0.001
Stringy pulse	58.921	<0.001

**Table 6:** Results of logistic regression analysis of 28 variables.

Variable	B	S.E.	Wald	Sig.	Exp(B)
Mental weakness	-1.917	0.405	22.440	<0.001	0.147
Languid	-2.305	0.693	11.061	0.001	0.100
Distortion of commissure	-1.386	0.503	7.582	0.006	0.250
Body emaciation	-3.114	0.640	23.657	<0.001	0.044
Limb without autonomous activity	-1.024	0.476	4.629	0.031	0.359
Thick fur	2.552	0.610	17.503	<0.001	12.828
Greasy fur	1.368	0.576	5.637	0.018	3.928

**Table 7:** Combined results of 6 index diagnostic tests.

No.	Combined Results
1	Thick fur
2	Greasy fur
3	Stringy pulse
4	Slippery pulse
5	Purplish tongue
6	Hesitant pulse
7	Thick fur, Greasy fur
8	Thick fur, Stringy pulse
9	Thick fur, Slippery pulse
10	Greasy fur, Stringy pulse
11	Greasy fur, Slippery pulse
12	Greasy fur, Purplish tongue
13	Stringy pulse, Slippery pulse
14	Stringy pulse, Purplish tongue
15	Slippery pulse, Purplish tongue
16	Thick fur, Greasy fur, Stringy pulse
17	Thick fur, Greasy fur, Slippery pulse
18	Thick fur, Stringy pulse, Slippery pulse
19	Greasy fur, Stringy pulse, Slippery pulse
20	Greasy fur, Stringy pulse, Purplish tongue
21	Greasy fur, Slippery pulse, Purplish tongue
22	Stringy pulse, Slippery pulse, Purplish tongue
23	Thick fur, Greasy fur, Stringy pulse, Slippery pulse
24	Greasy fur, Stringy pulse, Slippery pulse, Purplish tongue

**Table 8:** Evaluation results of 6 index diagnostic tests.

Variable	Se	Sp	Accuracy	YI	LR+	LR-	PV+	PV-
1	17.34	99.42	50.95	0.1676	29.8226	0.8314	97.73	45.48
2	83.06	72.67	78.81	0.5574	3.0398	0.2330	81.42	74.85
3	83.47	51.74	70.48	0.3521	1.7297	0.3195	71.38	68.46
4	91.53	59.30	78.33	0.5083	2.2491	0.1428	76.43	82.93
5	14.92	99.42	49.52	0.1434	25.6613	0.8558	97.37	44.76
6	0.81	75.58	31.43	-0.2361	0.0330	1.3124	4.55	34.57
7	9.68	99.42	46.43	0.0910	16.6452	0.9085	96.00	43.29
8	14.11	99.42	49.05	0.1353	24.2742	0.8639	97.22	44.53
9	15.73	99.42	50.00	0.1514	27.0484	0.8477	97.50	45.00
10	69.76	73.84	71.43	0.4360	2.6663	0.4096	79.36	62.87
11	79.84	75.00	77.86	0.5484	3.1935	0.2688	82.16	72.07
12	14.92	99.42	49.52	0.1434	25.6613	0.8558	97.37	44.76
13	78.23	59.88	70.71	0.3811	1.9500	0.3636	73.76	65.61
14	14.92	99.42	49.52	0.1434	25.6613	0.8558	97.37	44.76
15	14.92	99.42	49.52	0.1434	25.6613	0.8558	97.37	44.76
16	6.45	99.42	44.52	0.0587	11.0968	0.9410	94.12	42.43
17	8.87	99.42	45.95	0.0829	15.2581	0.9166	95.65	43.07
18	12.50	99.42	48.10	0.1192	21.5000	0.8801	96.88	44.07
19	66.53	75.58	70.24	0.4211	2.7247	0.4428	79.71	61.03
20	14.92	99.42	49.52	0.1434	25.6613	0.8558	97.37	44.76
21	14.92	99.42	49.52	0.1434	25.6613	0.8558	97.37	44.76
22	14.92	99.42	49.52	0.1434	25.6613	0.8558	97.37	44.76
23	5.65	99.42	44.05	0.0506	9.7097	0.9491	93.33	42.22
24	14.92	99.42	49.52	0.1434	25.6613	0.8558	97.37	44.76

**Table 9:** Glossary of TCM terminology.

TCM terminology	Description
Zang-fu	The Zang-fu organs are functional entities stipulated by Traditional Chinese Medicine. They constitute the centre piece of TCM's general concept of how the human body works. The term Zang included Heart, Liver, Spleen, Lung, Kidney; The term Fu included Small Intestine, Large Intestine, Gall Bladder, Urinary Bladder, Stomach and Sanjiao.
Qi	The ancient Chinese described Qi as “life force”. They believed it permeated everything and linked their surroundings together. Qi was also linked to the flow of energy around and through the body, forming a cohesive functioning unit. By understanding the rhythm and flow of Qi, they believed they could guide exercises and treatments to provide stability and longevity.
Phlegm-stasis	The Phlegm-stasis includes phlegm and blood stasis. The Phlegm is formed by the coagulation of body fluid. The Blood stasis refers to the poor functioning of the whole body, local blood stagnation and the presence of blood in the body.
Jing-luo	The meridians are believed to be channels running from the zang-fu in the interior of the body to the limbs and joints, transporting qi and xue. TCM identifies 12 “regular” and 8 “extraordinary” meridians.
Wind-evil	In the theory of TCM, Wind evil is a pathogenic factor. It is omnipresent. When the body's immunity decreases, it can invade the body and cause illness.

## Data Availability

The experiment data will not be shared as it involved privacy conditions.
